# Integrin-directed antibody-based immunotherapy: focus on VLA-4

**DOI:** 10.1093/immadv/ltab002

**Published:** 2021-02-09

**Authors:** Wilson Savino, Beatriz Chaves, Adriana Cesar Bonomo, Vinicius Cotta-de-Almeida

**Affiliations:** 1 Laboratory on Thymus Research, Oswaldo Cruz Institute, Oswaldo Cruz Foundation, Rio de Janeiro, Brazil; 2 National Institute of Science and Technology on Neuroimmunomodulation (INCT-NIM), Oswaldo Cruz Institute, Oswaldo Cruz Foundation, Rio de Janeiro, Brazil; 3 Rio de Janeiro Research Network on Neuroinflammation (RENEURIN), Oswaldo Cruz Institute, Oswaldo Cruz Foundation, Rio de Janeiro, Brazil; 4 Computational Modeling Group, Oswaldo Cruz Foundation, Eusébio, Ceará, Brazil

**Keywords:** cell trafficking, immunotherapy, neuroimmunology, T cells

## Abstract

One major finding of chronic inflammatory diseases of various origins is the establishment of inflammatory infiltrates, bearing different leukocyte subpopulations, including activated T lymphocytes. Integrins are among the large series of molecular interactions that have been implicated as players in both triggering and maintenance of leukocyte influx from the blood into a given organ parenchyme. Accordingly, blocking the interaction between VLA-6 integrin and laminin, experimentally abrogates heart graft rejection. Many reports have shown that VLA-4 is used by T cells to cross endothelial barriers, as well as to migrate within target tissues. In this respect, a humanized IgG4 anti-VLA-4 monoclonal antibody (specific to the α4-integrin chain of VLA-4) has been successfully applied to treat multiple sclerosis as well as inflammatory bowel disease. Anti-VLA-4 monoclonal antibody has also been applied to block transendothelial passage in other autoimmune diseases, such as rheumatoid arthritis. On this same vein is the action of such a reagent in impairing *in vitro* transendothial and fibronectin-driven migration of CD4^+^ and CD8^+^ T cells expressing high densities of VLA-4 from Duchenne muscular dystrophy patients, thus potentially enlarging the use of this strategy to other diseases. Yet, in a small number of patients, the use of Natalizumab has been correlated with the progressive multifocal leukoencephalopathy, a serious brain infection caused by the John Cunningham virus. This issue restricted the use of the reagent. In this respect, the development of smaller and more specific antibody reagents should be envisioned as a next-generation promising strategy.

## Introduction

A large series of molecular interactions have been implicated as players in triggering and maintenance of the influx of leukocytes, from the blood, through the blood vessel walls, into the given parenchyma. Integrins correspond to a large protein family of membrane receptors involved in this migratory path. They are integral cell membrane proteins; several of them being directly involved in cell migration. Additionally, a number of integrins able to bind to extracellular matrix moieties (e.g. laminin and fibronectin) are relevant for cell migration within tissues in a variety of organs. Lastly, integrin-directed interactions also play a role in the adhesion of leukocytes to cells in various organs, belonging or not to the hemopoietic system.

Chronic inflammatory diseases of various origins collectively correspond to a major public health issue, both in terms of social and economic consequences. One major common finding, in autoimmune and chronic inflammatory diseases, is the establishment of inflammatory infiltrates, bearing different leukocyte sub-populations, including activated T lymphocytes. Such infiltrates, harmful for the targeted tissue, can be either the origin of the illness, and/or play a significant role in the pathophysiological process perpetuating and providing a positive feedback to the disease.

Although steroids and other broad spectrum anti-inflammatory drugs are effective in treating a variety of inflammatory diseases, long-term usage has important side effects, such as bleeding, upper gastrointestinal complications, and opportunistic infections. Therefore, development of drugs that inhibit specific cellular functions without affecting normal immune surveillance is desirable. Herein, we will discuss selected examples, aiming at providing a discussion on the integrin-mediated immunotherapy, potentially applied as a further tool to treat inflammatory diseases as well as in survival of organ or tissue grafts.

Integrins are regarded as central adhesive molecules able to regulate the intricate pathway of leukocyte trafficking into tissues. They correspond to a large protein family of membrane receptors involved in this migratory path. For example, in a variety of tissues and organs, including the central nervous system (CNS), the pancreas and the skeletal muscle, inflammatory cells, particularly T lymphocytes, use the integrins to move toward and within the given tissue, as for example VLA-6 (α6β1, CD49f/CD29 – a laminin receptor) and VLA-4 (α4β1, CD49d/CD29 – which binds to VCAM-1 and fibronectin). In this respect, it is noteworthy that VLA-4 can be applied as a marker of T-cell activation in both humans and mice, being involved in leukocyte cytoskeleton dynamics [1–7]. Other integrins can direct the migration toward one specific tissue, as the integrin α4β7, which directs lymphocyte migration toward and within mucosal cell layers [4].

Consequently, blocking the establishment and/or maintenance of such a deleterious adhesive system in inflammatory reactions by using inhibitors of these integrins, should bring benefits to patients suffering from chronic inflammatory diseases, regardless their pathogenesis, including transplanted patients.

Consequently, blocking the establishment and/or maintenance of such a deleterious adhesive system in inflammatory reactions by using inhibitors of these integrins, should bring benefits to patients suffering from chronic inflammatory diseases, regardless their pathogenesis, including transplanted patients.

## Role of VLA-6-mediated interactions in graft rejection

The integrin VLA-6 (α6β1, CD49f/CD29) is a laminin receptor able to bind various laminin isoforms and plays a role in T-cell development, migration, and activation [8]. Previous studies strongly indicated that VLA-6 can be placed as a potential target to abrogate T-cell-mediated immune reaction, as for example graft rejection. Using implants of neonatal hearts into the subcutaneous tissue of the ears from adult syngeneic recipients, we showed that blocking VLA-6-mediated interactions with anti-VLA-6 prevented heart graft rejection by autoreactive spleen-derived CD4^+^ T lymphocytes obtained from mice previously infected with the parasite *Trypanosoma cruzi*, the causative agent of Chagas disease [9]. Similar data were observed when we applied an allogeneic transplant into normal adult recipients: both anti-laminin and anti-VLA-6 antibodies could prevent graft rejection [10, 11]. Moreover, specific blockade of the α5 laminin chain in the lymph nodes prevent activated cells to migrate from the lymph node toward the transplanted tissue, thus impacting graft survival [12].

These findings indicate that laminin/VLA-6-mediated infections can be envisioned as immunotherapeutic targets in controlling autoimmunity, as well as graft maintenance, not only at the rejection site, but also in lymph nodes. Yet, despite the consistent experimental data corresponding clinical assays have not yet been developed.

## Targeting VLA-4 as immunotherapeutic strategy to treat specific inflammatory diseases

As compared to VLA-6, much more data are available establishing VLA-4-mediated interactions as immunotherapeutic targets. VLA-4 is highly expressed in different activated T cell subsets; having vascular cell adhesion molecule-1 (VCAM-1, CD106), osteopontin, and fibronectin are natural cognate ligands. In this respect, it has been shown that CD49d-overexpressing T cell lines are autoreactive and proliferate in response to antigen-presenting cells, in an MHC class II-dependent manner, even in the absence of the cognate antigen [13].

VLA-4 is broadly expressed in cells of both innate and adaptive immune responses. Accordingly, this integrin is constitutively seen on the membranes of eosinophils and monocytes, as well as on T and B lymphocytes [14, 15]. Furthermore, it is expressed in all stages in intrathymic T-cell differentiation, particularly in the immature CD4/CD8 double-negative thymocytes [16].

**Figure 1:**
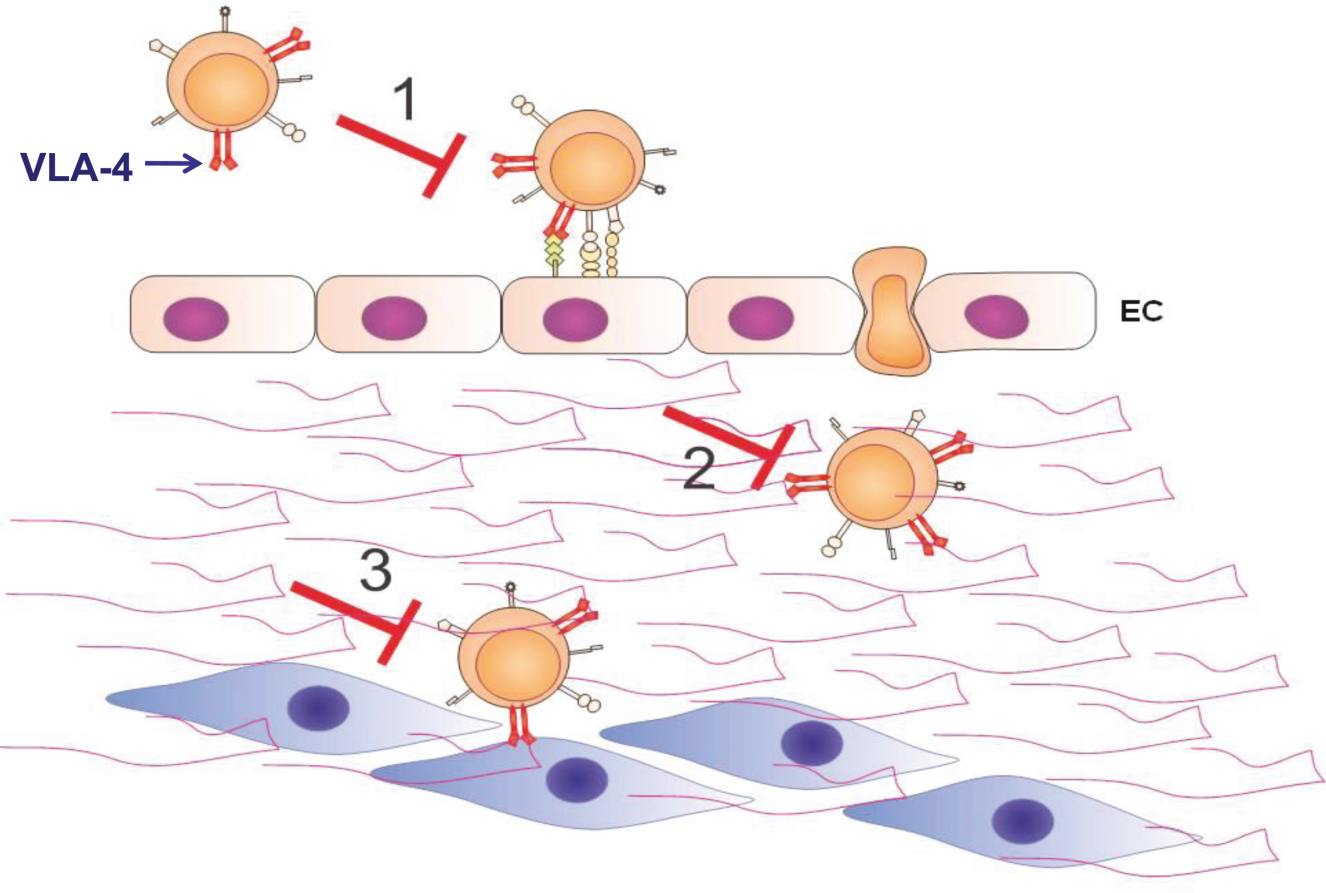
Anti-VLA-4 antibodies can potentially abrogate transendothelial and intra-tissular T cell migration and adhesion. 1. Extravasation of lymphocytes can be impaired by blocking VLA-4/VCAM-1 interaction at the endothelial of blood vessels; 2. Intratissular migration of lymphocytes in the inflammatory sites can be significantly diminished by blocking VLA-4/fibronectin interaction; 3. Adhesion of the activated lymphocyte to a potential organ specific cell type can be abolished by blocking fibronectin/VCAM-1/VLA-4 mediated cell–cell interaction. EC: endothelial cells; fibronectin is represented by the double waves in the extracellular space.

Additionally, VLA-4 overexpression in circulating T lymphocytes is associated with an increased in vitro adhesion to endothelial cells [3]. Accordingly (Fig. 1), blockade of VLA-4/VCAM-1 interaction should impair transendothelial migration of leukocytes to inflammation sites. Moreover, the blockade of VLA-4/fibronectin interaction should impair migration of leukocytes within the given target tissue, as well as binding to putative target cells. Of note, VLA-4 has an anti-apoptotic role in T lymphocytes [17]. In keeping with these findings, induction of apoptosis in lymphocytes has been shown as consequence of corresponding antibody therapy, in the model of autoimmune neuritis [18].

Therefore, biological products able to inhibit the alpha-4 integrin subunit represent potential immunotherapeutic agents to be applied in a larger spectrum of T-cell-related inflammatory diseases. Actually, since several years, large numbers of pre-clinical and clinical data strongly placed the integrin VLA-4 as a target for immunotherapy, particularly with the use of the anti- α4 integrin antibody [19–22].

Lastly, it is noteworthy that, as briefly mentioned above, the integrin α4β7, that binds to MAdCAM-1 (Mucosal Addressin Cell Adhesion Molecule-1), and also to VCAM-1 and fibronectin, is involved in lymphocyte migration to mucosal inflammatory sites, and has been a target for immunotherapy in gut inflammation [23, 24].

## Natalizumab: therapeutic role in multiple sclerosis and inflammatory bowel disease

Multiple sclerosis (MS) is a neurological autoimmune disease, being highly frequent in northern countries such as USA, Canada, and western European countries, and to a lesser extent in Latinoamerican and African countries, varying from less than 30 patients per 100,000 population in Brazil, to more than 150 per 100,000 in Canada. Additionally, it is more prevalent in females than males and represents the most common autoimmune disease in young adults. The disease has three main forms: relapsing and remitting MS, characterized by episodes of neurological dysfunction interspersed with periods of stability; primary-progressive MS, in which progressive neurological disability occurs from the outset; and secondary-progressive MS, in which progressive neurological disability occurs later in the course of the disease [25]. Axonal loss is the major determinant of the accumulation of irreversible (progressive) disability as a result of inflammation during both the relapsing and remitting and progressive phases of MS [26, 27].

MS is considered to be initiated by activated, self-reactive CD4^+^ T lymphocytes that recognize components of the myelin sheath, which surrounds and insulates nerve fibers. T cells enter the CNS through postcapillary venules and are reactivated by antigen-presenting cells in the perivascular space. These steps are followed by the recruitment of additional inflammatory cells, such as macrophages, which cause inflammation, edema and, eventually, destruction of the myelin sheath (28).

A largely used animal model for human MS is the experimental autoimmune encephalomyelitis (EAE), which can be induced in mice by immunization with myelin-derived peptide aa139-151. Animals develop clinical signs of disease, with intermittent episodes. This occurs with leukocyte infiltration within the CNS. Such an infiltration can be abrogated by anti-VLA-4 antibodies [19, 29–31]. Interestingly, not only VLA-4 is involved in the migration of T lymphocytes through the brain endothelium, but also in the recruitment of immature dendritic cells [32].

As mentioned above, beneficial effects of α4-integrin blockade were demonstrated in animal models as well as in clinical trials with MS patients. Based on these findings, the humanized whole monoclonal antibody Natalizumab [33], targeting the α4-integrin subunit, and that has been approved for treating relapsing-remitting MS. It has been demonstrated that Natalizumab was able to reduce the annual rate of MS relapse by 2/3 and to decrease the development of new gadolinium-enhancing lesions by ±90%, as ascertained by magnetic resonance imaging of MS patients [34]. Natalizumab is part of the therapeutic arsenal of drugs efficient against MS. Actually, a comparative study revealed that was not only more efficacious than fingolimod and dimethyl fumarate, but also was better tolerated by the patients [35]. Nonetheless, the use of Natalizumab has been correlated with the appearance of progressive multifocal leukoencephalopathy (PML), a serious and rare opportunistic infection of the brain caused by the John Cunningham virus (JCV). Since PML is a viral disease, such an adverse effect is likely to be due to the induction of immunodeficiency. These studies revealed that, despite the good general tolerability and sustained efficacy of Natalizumab for patients with severe MS, the risk of PML remained a concern [36]. At present, the use of Natalizumab has been restricted, as a monotherapy for MS patients presenting a highly active progressing disease in Europe and in the USA [37–40].

Natalizumab has also been applied in inflammatory bowel disease (IBD), a group of inflammatory conditions of the colon and small intestine of unknown etiology. The two major autologous types of IBD are Crohn’s disease (CD) and ulcerative colitis (UC), in which the immune system recognizes gastrointestinal tract moieties, causing what is considered an autoimmune inflammation. Treatment of CD comprises anti-inflammatory biologicals such as TNF antagonists, that target inflammatory pathways to induce remission in CD patients. However, approximately one third patients do not respond to anti-TNF therapy, and in some cases severe systemic side-effects have been reported [41]. In this context, Natalizumab has been applied to target a pathway other than TNF inhibition. The drug has demonstrated efficacy in inducing and maintaining remission in moderate-to-severe refractory CD patients with active inflammation [42]. However, due to possible occurrence of PML, the use of Natalizumab as a therapeutic strategy to treat CD is also rather restricted.

Migration to the intestines involves the presence of α4β7 and α4β1 integrins on the lymphocyte membrane [43, 44]. In this respect, it is noticeable that treatment with specific humanized anti-α4β7 antibodies revealed consistent good results in patients with UC [45, 23, 46, 47].

As α4β7 integrin is a central molecule for selective migration of T lymphocytes to the intestines [44], the treatment with specific humanized antibodies that target the interaction of MadCAM with α4β7 seems to overcome such a restriction. Interestingly, several clinical studies reported consistent good results in both UC and CD patients [47] and elicit new comprehensive studies on the migration-targeting immunotherapy of IBD [48].

## Potential use of anti-VLA-4 immunotherapy in other inflammatory diseases

### Rheumatoid arthritis

Rheumatoid arthritis (RA) is a systemic inflammatory disease affecting the joint lining tissue called synovium. The chronic character of autoimmune diseases has an important socio-economic impact. RA is the most frequent autoimmune disease with a prevalence of about 0.3 to 1% of the population worldwide and often associated with reduced mobility, increased social dependency, and finally work disability. RA patients are frequently at working age and the inability to work causes major financial and psychological issues for the person with the disease and their family. There is also the social and economic burden placed on the community resulting from a person’s incapacity to maintain employment.

RA patients are in general treated with a group of small molecular drugs called disease-modifying antirheumatic drugs (DMARDs). DMARDs suppress the body’s overactive immune and/or inflammatory systems in some way, thereby slowing down disease progression. RA patients not responding to DMARDs are treated with biological agents such as tumor necrosis factor (TNF) antagonists. Though TNF antagonists are effective in about two-thirds of the patients, the responding patients frequently become non-responsive within 5 years. Therefore, alternative treatments are required.

The synovium is normally a relatively acellular structure with a delicate intimal lining that is one or two cell layers deep. It covers the lubricating synovial fluid found in the cavities of synovial joints. The rheumatoid synovial tissue is characterized by hyperproliferation of fibroblast-like synoviocytes in the intimal lining layer and infiltration of the sublining by macrophages, T and B cells, which promote inflammation and destruction of bone and cartilage. Most leukocytes express VLA-4 on their surface and they interact with VCAM-1 expressed on synoviocytes and endothelial cells. Moreover, it has been shown that VLA-4/VCAM interactions between B lymphocytes and synovial fibroblasts upregulating expression of the anti-apoptotic protein Bcl-xL in B cells thus promoting B cell survival in the inflamed synovium [49]. Disruption of these VLA-4-mediated interactions between lymphocytes, synoviocytes and endothelial cells should put an end to the cycle of chronic inflammation, which is the hallmark of rheumatoid arthritis [50]. Accordingly, Natalizumab has been applied to treat RA patients. Nevertheless, a phase II, multicenter, double-blind, placebo-controlled clinical trial designed to determine the safety, tolerability and efficacy of Natalizumab in subjects diagnosed with moderate to severe RA receiving concomitant treatment with methotrexate. No statistically significant differences were found between Metothrexate-treated patients, in the presence or absence of Natalizumab [51]. Deeper investigation on mechanisms, as well as new tools should then be searched for.

### Potential use of anti-VLA-4 immunotherapy in Duchenne muscular dystrophy

**Figure 2:**
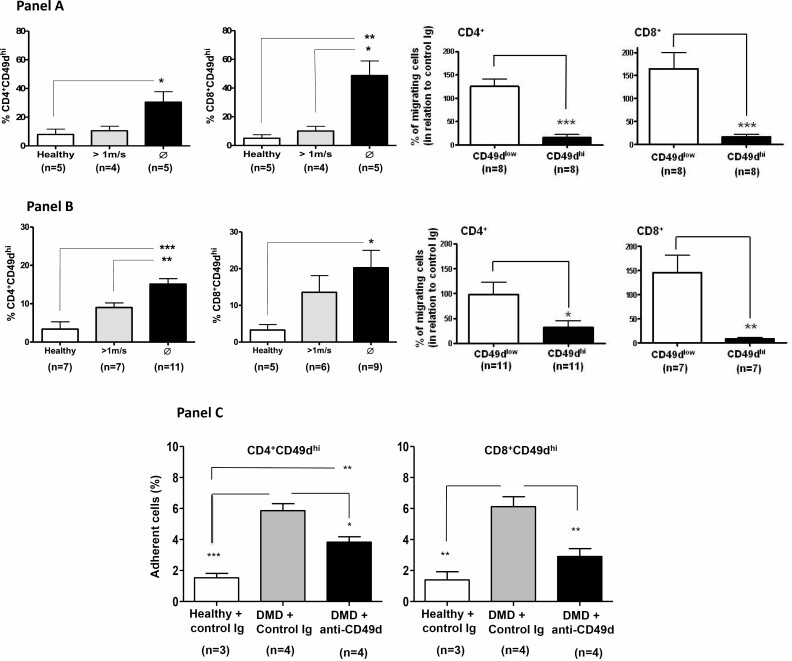
Role of VLA-4 in migration and adhesion of T-cells from Duchenne Muscular Dystrophy patients: blockade by anti-VLA-4 monoclonal antibody. Panel A reveals that transendothelial migration of CD4^+^ and CD8^+^ T cells expressing high densities of CD49d from DMD patients and unable to walk migrate more than the patients able to walk (upper graphics). Importantly, migration of CD49d^hi^ T cells is largely impaired in the presence of anti-VLA-4 antibody (bottom graphics). Similar enhancement of fibronectin-driven T cell migration is seen in panel B, which also shows that migration of CD49d^hi^ T cell subsets is largely impaired in the presence of anti-VLA-4 antibody. Finally, panel C provides evidence showing that both CD4^+^CD49d^hi^ and CD8^+^CD49d^hi^ T cells subsets adhere more to cultured human myoblasts, and that such an increase is abrogated by anti-VLA-4 antibody, as compared to unrelated Immunoglobulin. Groups were statistically compared using the Kruskal–Wallis test followed by Dunn’s multiple comparison test. *P < 0.05; **P < 0.01; ***P < 0.001. Modified from Pinto Mariz et al. 2015.

Muscular dystrophies are inherited diseases of the muscle that are characterized clinically by progressive muscle weakness, and pathologically by muscle degeneration. Among them, Duchenne muscular dystrophy (DMD) is the most common form of muscular dystrophy affecting 1 in 3500 newborn boys. DMD presents a progressive muscle weakness resulting in a loss of ambulation usually in the early teens and death around 30 years of age if modern standard care is applied. Despite the genetic cause for DMD, several studies in humans and animal models have suggested that the immune system is implicated in the pathophysiology of the muscular lesions [52–55]. We studied 74 DMD patients at different stages of disease and assayed for CD49d (the α4-integrin subunit) expression in circulating and intramuscular T-cells. Functionally, we tested transendothelial and fibronectin-driven migration, and adhesion to myotube monolayers. Increased percentages of circulating CD4^+^CD49d^high^ and CD8^+^CD49d^high^ T lymphocytes correlated with the more rapid disease progression. Moreover, CD49d^+^CD4^+^ and CD49d^+^CD8^+^ T cells were found in muscular inflammatory infiltrates. Importantly, T cells from severely affected patients exhibited higher transendothelial and fibronectin-driven migratory responses and increased adhesion to myotubes, when compared with control individuals [3]. As shown in Fig. 2, these responses were blocked with an anti-CD49d monoclonal antibody.

We also found increased numbers of circulating CD49^hi^CD4^+^ T lymphocytes in the Golden Retriever Muscular Dystrophy (GRMD) dog, a useful pre-clinical model for DMD, since it mimics the human disease in many aspects more closely than other existing mammalian models of dystrophin deficiency, including the classical mdx mouse. A significant increase was more important in rapid progressors as compared to slow progressors of the diseases. Similarly, CD49^hi^CD4^+^ T cells were present in the inflammatory infiltrates within the muscular tissue [56].

Overall, the data discussed in this item tell us that disease progression in DMD correlates with the increase in the relative numbers of CD49d^hi^ T cells (CD4 and CD8) in the blood. Moreover, increased numbers of CD49d^hi^ T cells (both CD4s and CD8s) predict a rapid progression of the disease. Additionally, CD49d expression on T lymphocytes can be used as a biomarker of disease progression in DMD (both in humans and in dystrophic dogs), including the stratification of patients before various clinical trials for other therapeutic strategies. Finally, VLA-4-directed interactions are potential therapeutic targets for selected patients in DMD aiming to improve their quality of life. Thus, the use of anti-VLA-4 antibodies is theoretically a promising approach to ameliorate the quality of life of DMD patients.

### Toward a second generation of anti-VLA-4 antibodies

Although the use of monoclonal antibodies as therapeutic tools is quite well established, most marketed antibodies are full-length humanized IgG molecules [57] that provide long half-lives and effector functions. However, there is a range of therapeutic applications in which other antibody formats may be more desirable. For instance, in some conditions, a long antibody serum half-life results in poor contrast in imaging applications, and inappropriate activation of Fc receptor-expressing cells may lead to massive cytokine release and associated toxic effects [58]. Furthermore, some IgG4 antibodies, as Natalizumab, may undergo half-antibody exchange *in vivo*, which can compromise the performance of the antibody even without damages on the clinical effects [59]. In addition, due to high molecular weight (~150 kDa), IgG antibodies are known to diffuse poorly into solid tissues and clear slowly from the body. By contrast, antibody fragments with specific antigen-recognition sites seem to be versatile stable, cost-effective, and efficient, therapeutic solutions for a range of autoimmune and potentially other inflammatory diseases [60].

Antibody fragments are, in general, less immunogenic due to the absence of Fc component of immunoglobulin [61]. This comprises a huge advantage in comparison to full-length antibodies, which induce the production of human anti-human antibodies (HAHAs) and therefore, activation of the immune system [62].

Besides their reduced immunogenicity, one of the main aspects of using antibody fragments is their ability to cross the blood–brain barrier (BBB) [63]. Most of the antibodies currently used for treating neurodegenerative diseases act mainly in the periphery, therefore, antibody fragments could be useful to perform directly in the CNS [64]. In addition, fragments can be applied to drug delivery, intracellular targeting, and labeling for imaging and diagnosis [64, 64]. In terms of cost-effectivity, it is well known that heterologous proteins produced by bacteria and yeast are cheaper, faster, and easier to produce comparing with mammalian, plant, and insect systems [65]. While full antibodies are recommended to be produced by mammalian cells, antibody fragments are produced in bacteria and yeast systems, are versatile and compatible with all the heterologous systems, which may reduce the costs of production by choosing bacteria as expression platform.

Considering that beneficial effects of blocking VLA-4 are evident, at least in severe MS and CD, it seems clear that novel α4-integrin blocking antibodies should be developed, particularly taking into that other autoimmune and chronic inflammatory diseases could benefit from such treatment. Therefore, smaller antibody molecules such as the antigen-binding fragment (Fab) or the variable fragment (Fv) should be envisioned as further anti-VLA-4 therapeutic agents [66–68]. Single-chain Fv (scFv) molecules are fragments of antibodies composed of the VH and VL domains of the corresponding immunoglobulin, joined by a flexible linker peptide of variable size. These proteins have an average molecular weight of 30 kDa [69], and have two disulfide bridges, one related to VH and the other to VL sequences. Because scFvs are formed by variable domains, they have all six CDRs that make up their antigen recognition region [70]. In addition to the composition, the size of the binding peptide is fundamental to the scFv molecule. Studies have shown that when comparing different scFvs made up of ligand peptides of varying sizes, the reactivity and specificity properties of scFvs have been can change [71]. Therefore, not only the CDRs of these antibody fragments influence the affinity and specificity of scFv, but also its structural conformation and type of peptide linker.

In this context, we recently produced a scFv antibody fragment able to specifically recognize VLA-4 (pending patent deposited at the Brazilian National Institute of Intelectual Property – INPI –, number BR 10 2020 016890 8). The scFv nucleotide sequence was previously designed by using *in silico* tools as database search, molecular modeling and docking, site-directed mutagenesis, and molecular dynamics. Docking results showed that the scFv presented favorable parameters, namely Haddock Score, Cluster size and RMSD, for its interaction with VLA-4, as compared with two other integrins (LPAM-1 and VLA-5). Molecular dynamics confirmed the docking results and further showed that the main interactions involved are salt bridges, electrostatic and Van der Waals interactions. The scFv sequence thus obtained was cloned and expressed in *Escherichia coli*. Functionally, this scFv antibody was able to significantly impair the adhesion of T cells (Jurkat T cell line), on surfaces coated with VCAM-1 (Table 1). Furthermore, experiments performed under flow conditions showed that the scFv reduced the adhesion frequency of primary T lymphocytes over VLA-4 ligands. This scFv product also interfered with the pattern of distribution of actin and phosphotyrosine in CD8^+^ T cells activated by anti-CD3 and fibronectin. Overall, this reagent seems promising, although it still needs validation in relevant pre-clinical models of selected autoimmune and chronic inflammatory diseases is necessary.

**Table 1:** Migration of Jurkat T cells over *transwell chambers* coated with VCAM-1: blockage by Natalizumab and anti-VLA-4 scFv antibodies

Coating ligand^a^	Cell treatment^b^	Number of transmigrating cells^c^	*P* value: versus control^d^	*P* value: versus natalizumab^d^
VCAM-1	None	30.41 ± 13.25	-----	**0.0058**
VCAM-1	Anti VLA-4 scFv	9.50 ± 4.24	**0.0440**	0.582
VCAM-1	Natalizumab	1.92 ± 0.29	**0.0058**	-----
BSA	None	3.10 ± 3.10	**0.0073**	0.993

^a^VCAM-1 concentration = 2.5 µg/ml; BSA concentration: 2 µg/ml; ^b^Anti VLA-4 scFv and Natalizumab concentration = 20 µg/ml; ^c^Cell numbers × 10^3^ ± standard deviation. Means of three independent experiments. ^d^Dunnett’s multiple comparisons test. Statistically significant *P* values are shown in bold.

## Concluding remarks

Although steroids and other anti-inflammatory drugs with broad-spectrum activities are effective in treating a variety of inflammatory diseases, long-term usage is known to have unacceptable side effects, such as greater risk of bleeding, upper gastro-intestinal complications, and infection caused by alteration of phagocytic leukocyte migration and function. Therefore, in the treatment of chronic inflammatory diseases, it is desirable to develop drugs that inhibit more selectively specific cellular functions without affecting normal immune surveillance.

The data summarized above provide evidence showing that targeting VLA-4, by applying humanized anti-VLA-4 antibody was a relevant therapeutic strategy to treat at least severe refractory inflammatory diseases. Nevertheless, a new generation of inhibitors will certainly be welcome, and the development of smaller and more selective antibodies should be envisioned. In this respect, the design of similar molecules, but only containing the single chain variable fragment of the α4 integrin chain seems to be a promising strategy.

Using antibody fragments may overcome some limitations related to full anti-VLA-4 antibodies. The absence of Fc portion avoids unnecessary immune system activation and allows penetration across the BBB, which could increase the antibody performance directly within the CNS. In addition, antibody fragments can be cleared from the body faster than full antibodies, which may be relevant to prevent harmful effects related to permanent VLA-4 blockade.

Specificity for such a reagent could be further improved by constructing double-specific scFVs directing the anti-integrin to target specific cells.

Having said that, it should be pointed out that VLA-4 is not the only α4-containing integrin that deserves more research and technology improvement. As mentioned above, target α4β7 integrin using the same antibody strategy has been proven to be efficient in gut associated autoimmune diseases.

Lastly, other integrin-directed cellular interactions should be better investigated, so that to enlarge the possibility of reagents to tackle specific and potentially harmful activated T lymphocytes in chronic inflammatory diseases and controlling organ transplantation.

## Data Availability

No new data were generated or analysed in support of this research.

## Abbreviations

BBB: Blood–brain barrier
CD: Crohn’s disease
DMARDs: Disease-modifying antirheumatic drugs
DMD: Duchenne muscular dystrophy
Fab: Antigen-binding fragment
Fv: Variable fragment
IBD: Inflammatory bowel disease
MS: Multiple sclerosis
PML: Progressive multifocal leukoencephalopathy
RA: Rheumatoid arthritis
scFv: Single-chain Fv
TNF: Tumor necrosis factor
UC: Ulcerative colitis
VH: Variable portion of the heavy chain of immunoglobulin
VLA: Very late antigen
VL: Variable portion of the light chain of Immunoglobulin
